# Intercultural Profiles and Adaptation Among Immigrant and Autochthonous Adolescents

**DOI:** 10.5964/ejop.v11i1.872

**Published:** 2015-02-27

**Authors:** Cristiano Inguglia, Pasquale Musso

**Affiliations:** a Department of Psychological, Educational and Training Sciences, Università degli Studi di Palermo, Palermo, Italy; Academy of Special Education, Warsaw, Poland

**Keywords:** acculturation, immigrants’ adaptation, multiculturalism, person-centred approach, adolescence, profiles

## Abstract

Few studies examine relationships between intercultural strategies and adaptation among adolescents using a person-oriented approach. Framed from an intercultural psychology perspective, this study used such an approach in order to examine the influence of intercultural profiles, patterns of relationships among variables related to intercultural strategies, on the adaptation of adolescents of both non-dominant and dominant groups. Two hundred and fifty-six adolescents living in Italy and aged from 14 to 18 participated to the study: 127 immigrants from Tunisia (males = 49.61%) and 129 autochthonous (males = 44.19%). Data were collected through self-report questionnaires. Using cluster analytic methods to identify profiles, the results showed that immigrant adolescents were divided in two acculturation profiles, ethnic and integrated-national, with adolescents belonging to the latter showing higher self-esteem, life satisfaction and sociocultural competence than the former. Also among autochthonous adolescents two acculturation expectation profiles were identified, not-multicultural and multicultural, with adolescents belonging to the latter showing higher self-esteem and life satisfaction than the former. Findings highlight the importance of using multiple indicators in order to gain a more comprehensive understanding of the acculturation process as well as suggesting implications for the social policies in this field.

Italy is facing a rapid enlargement of immigrant population. From the second half of the 1970s the country has witnessed a solid influx of immigrants which have increased with a high rate of growth. In fact the total amount of legal immigrants who are permanent residents in Italy has changed from about 1,000,000 in 2001 to about 5,000,000 in 2013 (Caritas Italiana & Fondazione Migrantes, 2013).

This situation results in a patchwork of religions, people and cultures that requires innovative social policies in order to promote the intercultural dialogue and to overcome conflicts between groups, especially during a period of economic recession characterized by high unemployment rates and social dissatisfaction. Psychology and social sciences can effectively contribute to an understanding of those factors which foster personal and societal development in multicultural contexts (Berry, 2005, 2013).

Intercultural psychology has contributed to this understanding through two distinct approaches, named acculturation and ethnic relations (Berry, 2005, 2006). The first approach has been concerned with the views held by non-dominant groups (e.g., immigrants) regarding how they wish to live in the host society, using such concepts as acculturation strategies and adaptation. The second approach has examined the views and behaviours of the dominant group toward the non-dominant ones, using concepts such as acculturation expectations. However, both the approaches represent “one-way” views of intercultural relations which miss examining the reciprocal views held by non-dominant groups towards dominant group, and vice versa.

In the current study we attempted to bring together the two approaches and to examine concurrently both non-dominant and dominant groups following the guidelines developed by Berry in the framework of the international collaborative project Mutual Intercultural Relations in Plural Societies (MIRIPS)^i^. The findings presented below come from the Italian section of MIRIPS and refer to adolescents, who are in a critical developmental period for the formation of acculturation processes (Sam & Berry, 2006).

The paper is focused on the concept of intercultural strategies that designates individuals’ and groups’ preferences towards the ways in which they wish to engage with their own and other cultural groups (Berry, 2013). They are referred both to non-dominant and dominant groups: in the first case they are called acculturation strategies, in the second case they are defined acculturation expectations (Berry, 2003, 2005). The impact of intercultural strategies on adaptation of immigrant and autochthonous adolescents has been already analyzed by several authors (Berry, Phinney, Sam, & Vedder, 2006a, 2006b; Brown & Zagefka, 2011; Giang & Wittig, 2006; Güngör, 2007; Ljujic, Vedder, Dekker, & van Geel, 2012; Motti-Stefanidi, Berry, Chryssochoou, Sam, & Phinney, 2012; Nguyen & Benet-Martínez, 2013). However, these studies reveal the prevalent use of a variable-oriented approach (Magnusson & Bergman, 1988) centred on intercultural strategies without seeing at the individual in his/her totality. This approach focuses the analysis on variables and assumes that they are interrelated similarly and linearly across all groups under consideration (McQuitty, 1987). Therefore, the variable-centred approach supposes that the meaning and covariation of all variables is the same for all groups (Bergman, Cairns, Nilsson, & Nystedt, 2000). Although the studies that used this approach have revealed a great deal of information in the field of intercultural psychology, immigrant and autochthonous groups also exhibit patterns of several interrelated dimensions that are not captured by unidimensional analyses (Berry et al., 2006b). Hence, we used a holistic person-oriented approach that provides information concerning which combinations of factors related to intercultural strategies are significant and prevalent, and how such combinations may change for different subgroups of adolescents (Bergman & Trost, 2006; Magnusson, 2003). We first created a classification of adolescents based on several dimensions related to intercultural strategies, then we examined the multidimensional relationships among dimensions (patterns). These patterns are here defined as intercultural profiles and consist of acculturation profiles with regard to non-dominant groups, and acculturation expectation profiles with regard to dominant groups. Also, such an approach allows us to determine whether the influence of isolated intercultural variables (e.g., intercultural strategies), on adolescents’ adaptation keeps its actuality when we consider multivariable systems as intercultural profiles.

Starting from these considerations the paper tried to answer three critical questions in this field: a) What are the acculturation profiles of immigrant adolescents? b) What are the acculturation expectation profiles of autochthonous adolescents? c) How are these intercultural profiles related to adaptation of both immigrant and autochthonous adolescents?

## From Acculturation Strategies to Acculturation Profiles

Acculturation strategies are a central concept in the well-known Berry’s model of acculturation (Berry, 1997) which considers two basic dimensions as important for the adaptation of immigrants: the preference for maintenance of the own ethnic culture (cultural maintenance) and the preference for involvement or contact with the host society (cultural contact). By combining these dimensions, four acculturation strategies can be distinguished: integration (high maintenance and high contact), assimilation (low maintenance and high contact), separation (high maintenance and low contact), and marginalisation (low maintenance and low contact).

The role of acculturation strategies in affecting immigrant adolescents’ adaptation has been already analyzed by several studies showing that integration is the most adaptive outcome, whereas marginalization the worst (Berry et al., 2006a, 2006b; Giang & Wittig, 2006; Güngör, 2007; Motti-Stefanidi, Pavlopoulos, Obradović, & Masten, 2008; Neto, 2002; Nguyen & Benet-Martínez, 2013). However, some authors (Berry et al., 2006b; Brown, Gibbons, & Hughes, 2013; Brown & Zagefka, 2011; Rudmin, 2009; Schwartz, Unger, Zamboanga, & Szapocznik, 2010) have suggested to move from a variable-oriented to a person-oriented approach (Bergman & Magnusson, 1997) in order to identify some patterns of acculturation. In this perspective, the use of empirical ways of classifying individuals, such as cluster or latent class analysis, with acculturation data is seen as fundamental because clustering methods permit a more holistic approach to acculturation in order to identify more realistic profiles with respect to the acculturation outcomes (e.g., Brown et al., 2013; Schwartz & Zamboanga, 2008).

For instance, Berry et al. (2006b) carried out a cluster analysis with factors associated to the acculturation process, such as acculturation strategies, ethnic and national identities, language use, ethnic and national peer social contacts, on data collected in the ICSEY (International Comparative Study of Ethnocultural Youth) project that are referred to immigrant adolescents living in different countries (e.g., Australia, Canada, France, Israel, New Zealand, Portugal, and USA). Following this procedure they have identified four acculturation patterns or profiles named *integration*, *national*, *ethnic*, and *diffuse*.

Adolescents who belong to the *integration* profile (36.40% of the sample) are characterized by a positive orientation towards both original and host society cultures with high levels of both ethnic and national identities, frequent social contacts with people who belong to both their own group and to the host society, and low levels of assimilation, separation, and marginalisation. Adolescents who belong to the *national* profile (18.70%) are characterized by a strong orientation toward the host society with high levels of national identity, assimilation, social contacts with members of the national group, and low levels of ethnic identity. Adolescents who belong to the *ethnic* profile (22.50%) show a strong orientation towards their own group with high levels of ethnic identity, separation, and social contacts with members of their ethnic group, together with low levels of assimilation and national identity. Finally, adolescents who belong to the *diffuse* profile (22.40%) are uncertain about their role in the host society, showing high proficiency in the ethnic language, high levels of assimilation, marginalisation, separation, low ethnic identity, low national identity and social contacts with peers of the host country.

Other studies that employed similar methods have revealed more complex results that only partly coincide with the four acculturation strategies. For instance, Schwartz and Zamboanga (2008) conducted a latent class analysis on scores related to acculturation strategies, orientation toward heritage and American cultural practices in areas such as language use and entertainment among Hispanic college students living in Miami. They identified six classes corresponding to acculturation profiles. In particular, two of them resembled variants of integration (named *Full bicultural* and *Partial bicultural*), other two resembled a combination of assimilation and integration (*American oriented bicultural* and *Assimilated*), one resembled a combination of separation and integration (*Separation*), and one (*Undifferentiated*) that did not remind any of Berry’s typologies. The clusters characterized by a combination of assimilation and integration were the most frequent in the sample considered in the study.

Moreover, Brown et al. (2013) conducted a cluster analysis using five clustering variables: (1) heritage practices, (2) U.S. practices, (3) heritage attitudes, (4) U.S. attitudes, and (5) heritage identity. Following this approach the authors found four clusters among immigrant university students living in US. One cluster, labelled *U.S. Practices*, was similar to assimilation, whereas cluster labelled *Heritage Practices* closely resembled the category of separation. The other two clusters, labelled *Bicultural Attitudes*, and *Bicultural Practices & Heritage Identity*, were similar to integration (i.e., biculturalism). None of the clusters resembled marginalization.

Taken together, these results highlight that not all of Berry’s categories may exist in a sample or population, as stated by Schwartz et al. (2010). In particular, some studies did not reveal a marginalization cluster (e.g., Brown et al., 2013; Schwartz & Zamboanga, 2008). For many immigrant people, especially attending high schools or colleges, marginalization does not seem a relevant category.

## From Acculturation Expectations to Acculturation Expectation Profiles

According to Phinney, Berry, Vedder, and Liebkind (2006), it is also interesting to look at the intercultural strategies of the dominant group in the society of settlement which are better known as acculturation expectations. They refer to the views of autochthonous people about how immigrants should acculturate.

The acculturation expectations are usually identified by asking autochthonous people whether immigrants should maintain their cultural attributes and/or seek contact with other cultural groups. On the basis of these two dimensions, four acculturation expectations can be derived: segregation (when autochthonous wish immigrants’ separation), multiculturalism (when autochthonous wish immigrants’ integration), melting pot (when autochthonous wish immigrants’ assimilation), and exclusion (when autochthonous wish immigrants’ marginalization).

The acculturation expectations were examined in different surveys across the world (Berry, 2011, 2013). However, the findings of some studies indicated that, rather than there being four distinct acculturation expectations, they could represent a unidimensional construct, with preference for multiculturalism anchoring one end of the dimension, and the other expectations anchoring the other end.

Although several authors have focused their interest on these expectations (Berry, 2006; Bourhis, Moïse, Perreault, & Senécal, 1997; Ljujic et al., 2012), there is a lack of studies that, similarly to immigrants’ acculturation profiles presented above, attempt to identify specific patterns of acculturation expectations (here named *acculturation expectation profiles*) using a person-oriented approach with variables involved in the process of multicultural acceptance (e.g., acculturation expectations, national identity, social contacts with people from national group and from other cultural groups).

## Relationships Between Intercultural Profiles and Adolescents’ Adaptation

Few studies deal with the relationships between intercultural profiles and adaptation. Most of them are focused only on immigrants and investigate the relationships between acculturation profiles and adaptation of immigrant adolescents (e.g., Berry et al., 2006b; Brown et al., 2013). In these studies two forms of adaptation were considered: psychological and sociocultural adaptation (Searle & Ward, 1990). The former refers to mental and physical well-being, whereas the latter emphasizes immigrants’ success in effectively organizing their daily lives in the new context (e.g., in terms of acquiring facility with the new language, gaining cultural knowledge, and establishing a network of social relationships).

Berry and colleagues (2006b) have found that immigrant adolescents who belong to the integration profile show higher levels of psychological and sociocultural adaptation than the other profiles, whereas adolescents who belong to the diffuse profile display the lower levels of psychological and sociocultural adaptation than the other profiles. Moreover, adolescents who belong to the national profile show relatively low levels of psychological adaptation in terms of self-esteem, life satisfaction and psychological problems, whereas they were not clearly distinct from other profiles with respect to sociocultural adaptation. Finally, adolescents who belong to the ethnic profile show high levels of psychological adaptation but low levels of sociocultural adaptation, showing some difficulties in organizing their daily lives in the new context. However, these results need to be confirmed by other studies involving adolescents living in different countries from those considered by these authors. Also Brown et al. (2013) found that immigrants who belonged to either of the clusters resembling integration (Bicultural Attitudes, or Bicultural Practices & Heritage Identity) reported significantly higher life satisfaction than the participants who belonged in the cluster that resembled separation (Heritage Practices). These findings are concordant with Berry’s integration hypothesis (Berry, 2013) stating that individuals feel well and do well if they are engaged in both their own culture and that of the larger society.

With regard to the autochthonous adolescents, there are no studies aimed at analyzing the relationships between acculturation expectation profiles and adaptation. However, some authors have found that a positive attitude towards multiculturalism is often associated with high levels of psychological well-being, especially for self-feelings, among adolescents (Verkuyten, 2009; Verkuyten & Thijs, 2004). For example, Verkuyten and Thijs (2004) have observed that multiculturalism has a positive effect on self-esteem of both ethnic minority and majority adolescents living in the Netherlands. In a similar way, Verkuyten (2009) has found that the self-feelings of the Dutch adolescents were positively associated with their attitude towards the cultural diversity and recognition for all groups in society.

## Aims and Hypotheses

The general aim of the present study was to analyze emerging intercultural profiles in relation to the adaptation of immigrant and autochthonous adolescents living in Sicily (Italy). In particular, the study has four specific goals. The first goal was to identify some acculturation profiles among Tunisian adolescents on the basis of their scores of acculturation strategies, ethnic and national identities, ethnic and national peer social contacts. In accordance with Berry and colleagues (2006b), we expected to identify four acculturation profiles: integration, national, ethnic, and diffuse.

The second goal was to identify some acculturation expectation profiles among autochthonous adolescents on the basis of their scores of acculturation expectations, national identity, ethnic and national peer social contacts. Berry (2011) argues that in contexts characterized by a positive attitude towards multiculturalism it is likely to observe an unilinear structure of the acculturation expectations with multiculturalism at one end of the dimension, and the other expectations at the other end. Considering that Sicilian children and adolescents are generally characterized by positive attitudes towards other national groups (Inguglia & Musso, 2013), we hypothesized to find two cluster, one resembling multiculturalism and the other including a combination of higher rates of melting pot, exclusion and segregation.

The third goal of this study was to analyze the relationships between acculturation profiles and psychological and sociocultural adaptation of immigrant adolescents. On the basis of the integration hypothesis (Berry, 2013), we expected that adolescents who belong to the integration profile would show higher levels of psychological and sociocultural adaptation than the other profiles, whereas those who belong to the diffuse profile would display lower levels of adaptation than the other profiles.

Finally, the fourth goal was to investigate the relationships between the acculturation expectation profiles and the psychological adaptation of autochthonous adolescents. Following the suggestions from the literature on this topic we expected to find an association between the membership to a profile characterized by positive attitude towards multiculturalism and positive adaptation, especially in terms of self-esteem.

## Method

### Participants

As already mentioned in the introduction, data came from the Italian section of MIRIPS project. Data about immigrant adolescents referred to Tunisian group that was chosen because it is one of the most prominent non-dominant groups in the research context, that is Sicily. Tunisian community is characterized by strong ties with the heritage culture that are facilitated by the presence of many resources available, such as Tunisian primary schools, places of worship, community centres, and typical restaurants. Despite the existence and the proximity of these resources, many Tunisians are also interested in having good relationships with Sicilian people (Inguglia & Lo Coco, 2009).

The total group of participants consisted of 256 Tunisian and Italian adolescents aged from 14 to 18 years, living in western Sicily (Italy) in towns with more than 50,000 inhabitants. Tunisian adolescents were 127 (males = 49.61%; mean age = 15.64, *SD* = 1.19). The group included both first-generation (those who were born in the country of origin and arrived after the age of 6 years) and second-generation immigrants (those who were born in Italy, or arrived before the age of 6 years). First-generation adolescents were 54 (males = 51.85%; mean age = 15.72, *SD* = 1.17). All of them were born in their country of origin, had arrived in Italy at an average of 11.75 years (*SD* = 2.67), and had lived in Italy for an average of 3.97 years (*SD* = 2.45). Second-generation adolescents were 73 (males = 47.95%; mean age = 15.58, *SD* = 1.20). The majority (53.42%) of them were born in Italy; adolescents who were not Italy-born had arrived in Italy at an average of 2.20 years (*SD* = 1.73) and had lived in Italy for an average of 13.83 years (*SD* = 1.65). About eighty percent of immigrant youngsters attended a secondary school, while about 20.00% were workers. All of them were living in one household with their parents and 95.28% of them came from intact two-parents families, 3.94% had divorced or separated parents, and 0.78% came from a family in which one of the parents had died. Socio-economic status (SES) of their families was prevalently low; based on a three-stratum classification of scores from the Barratt Simplified Measure of Social Status (BSMSS, Barratt, 2006), 78.74% fell into the low stratum, 13.29% into the medium, and 7.97% into the high. Italian adolescents were 129 (males = 44.19%; mean age = 15.92, *SD* = 1.46). They were randomly selected in the same schools and neighbourhood of the Tunisian adolescents. About ninety percent of Italian youngsters attended a secondary school, while about 10.00% were workers. All of them were living in one household with their parents and 83.72% of them came from intact two-parents families, 14.73% had divorced or separated parents, and 1.55% came from a family in which one of the parents had died. SES of their families was prevalently medium; 24.81% fell into the low stratum, 53.49% into the medium, and 21.70 into the high.

### Procedure and Measures

The study, as part of the MIRIPS project, was approved by the local Psychology Department’s ethics committee and was performed in accordance with the Italian Association of Psychology ethical principles for psychological research. Data collection involved completion of two structured versions of MIRIPS questionnaire, one for immigrant groups and one for the host group (Annis, Gibson, & Berry, 2010). All participants were contacted in schools as well as in community centres. After having received permission from the respective principals, participants’ parents were informed about the purpose of the research, the voluntariness of participation and the anonymity of responses through specific parent meetings. During the meetings, parents provided informed consent for their son’s or daughter’s participation. Less than 5% of the parents did not allow their son or daughter to participate. Nonetheless, adolescent participants provided signed assent agreeing to participate, also. The data were collected by Italian research assistants and young graduate trainees. Questionnaires were single-administered with the support of two cultural mediators when needed for the Tunisian group. Both versions of the questionnaire assessed a wide range of variables. For the goals of this paper, only some measures were taken into account. These measures were successfully used in other relevant studies (e.g., Berry et al., 2006b; Lebedeva & Tatarko, 2013; Robinson, 2009; Schmitz & Berry, 2009) and were adapted in different countries, including Italy (Berry, 2014).

#### Demographic Variables

Respondents were asked to indicate their gender (0 = female; 1 = male), age, state of birth, age of arrival in the country of residence, and current occupation. Using the information about the state of birth and the age of arrival in the country of residence, we constructed a generation variable (0 = first-generation; 1 = second-generation) and a length of residence variable, used both as a continuous variable and as a categorical variable (1 = 0-6 years, 2 = 6-12 years, and 3 = 12-18 years). Family characteristics, number of parents, paternal and/or maternal level of school completed and their occupation were assessed using BSMSS (Barratt, 2006).

#### Immigrants’ Acculturation Attitudes

A 16-item scale assessed four acculturation attitudes: assimilation, integration, separation, and marginalisation (Berry et al., 1989). The items concern four domains of life of non-dominant group adolescents: cultural traditions, language, social activities, and friends. For example, the items in the social activities domain include four questions: “I prefer social activities which involve both Italians and Tunisians” (integration); “I prefer social activities which involve Italians only” (assimilation); “I prefer social activities which involve Tunisians only” (separation); and “I don’t want to attend either Italians’ or Tunisians’ social activities” (marginalisation). The items were presented as declarative statements and participants were asked to indicate on a 5-point scale (from 1 = *very untrue* to 5 = *very true*) the extent to which each statement was true for them. In the present study, the subscales had adequate internal consistency with Cronbach’s α ranged from .63 to .76.

#### Host Group’s Acculturation Expectation

A 16-item scale (Berry, 2003) assessed four acculturation expectations: multiculturalism, melting pot, segregation, and exclusion. The items concern four domains of life of dominant group adolescents: cultural traditions, language, social activities, and friends. For example, the items in the social activities domain include four questions: “I feel that Tunisians should engage in social activities that involve both Italians and their own group” (multiculturalism); “Tunisians should engage in social activities that involve their own group members only” (segregation); “Tunisians should engage in social activities that involve Italians only” (melting pot); and “Tunisians should not engage in either Italian or their own group’s social activities.” (exclusion). The items were presented as declarative statements; participants were asked to indicate on a 5-point scale (from 1 = *very untrue* to 5 = *very true*) the extent to which each statement was true for them. In the present study, the subscales had adequate internal consistency with Cronbach’s α ranged from .62 to .74.

#### Cultural Identity

Immigrants’ ethnic identity was measured with an 7-item scale assessing ethnic affirmation (e.g. sense of belonging, positive feelings about being group member) of non-dominant group adolescents (Phinney, 1992). A sample item is “I feel that I am part of Tunisian culture”. The items were presented as declarative statements; participants were asked to indicate on a 5-point scale (from 1 = *very untrue* to 5 = *very true*) the extent to which each statement was true for them. In the present study, Cronbach’s α was .82. National identity was assessed with a 3-item scale assessing national affirmation and the importance of one’s national identity of non-dominant and dominant group adolescents (Phinney & Devich-Navarro, 1997). A sample item is: “I am happy that I am Italian”. The items were presented as declarative statements; participants were asked to indicate on a 5-point scale (from 1 = *very untrue* to 5 = *very true*) the extent to which each statement was true for them. In the present study, Cronbach’s α was .90 and .87 for non-dominant and dominant group adolescents respectively.

#### Ethnic Peer Contact

Two items assessed the number and the frequency of contacts with Tunisian peers for non-dominant, and with ethnic minority peers for dominant, group adolescents (Berry et al., 2006b). The items are respectively: “How many close Tunisian (or ethnic minority) friends do you have?” and “How often do you meet with?”. Participants responded on a scale ranging from *none* (1) to *many* (5) in the former case and from *never* (1) to *daily* (5) in the latter case. The ethnic peer contact was measured as a composite variable by extracting the square root of the product of the two items.

#### National Peer Contact

Two items assessed the number and the frequency of contacts with Italian peers for non-dominant and dominant group adolescents (Berry et al., 2006b). The items are respectively: “How many close Italian friends do you have?” and “How often do you meet with?”. Participants responded on a scale ranging from *none* (1) to *many* (5) in the former case and from *never* (1) to *daily* (5) in the latter case. The ethnic peer contact was measured as a composite variable by extracting the square root of the product of the two items.

#### Psychological Adaptation

It was measured with three scales assessing self-esteem, life satisfaction, and psychological problems. Self-esteem was measured with the Rosenberg Self-Esteem Scale (Rosenberg, 1965; Italian adaptation by Prezza, Trombaccia, & Armento, 1997) which consists of 10 items. A sample item is “On the whole I am satisfied with myself”. The items were presented as declarative statements and participants were asked to indicate on a 5-point scale (from 1 = *very untrue* to 5 = *very true*) the extent to which each statement was true for them. In the present study, Cronbach’s α was .77 and .79 for non-dominant and dominant group adolescents respectively. Life satisfaction was measured with a 5-item scale which assessed the overall degree of adolescents’ satisfaction with their lives (Diener, Emmons, Larsen, & Griffin, 1985). A sample item is: “I am satisfied with my life”. The items were presented as declarative statements and participants were asked to indicate on a 5-point scale (from 1 = *very untrue* to 5 = *very true*) the extent to which each statement was true for them. In the present study, Cronbach’s α was .82 and .84 for non-dominant and dominant group adolescents respectively. Psychological problems were measured with a 15-item scale assessing depression, anxiety, and psychosomatic symptoms (Berry et al., 2006b). A sample item is: “My thoughts are confused”. The items were presented as declarative statements and participants were asked to indicate on a 5-point scale (from 1 = *very untrue* to 5 = *very true*) the extent to which each statement was true for them. In the present study, Cronbach’s α was .88 and .90 for non-dominant and dominant group adolescents respectively.

#### Immigrants’ Socio-Cultural Competence

It was measured with a 20-item scale (Ward & Kennedy, 1999) assessing difficulty for non-dominant group adolescents to live in the host country. It focuses on the skills needed to cope with everyday social situations encountered in a new culture. A sample item is: “Please indicate how much difficulty you experience living here in Sicily in each of these areas: e.g., Making friends”. Participants were asked to indicate on a 5-point scale (from 1 = *no difficulty* to 5 = *many difficulties*) the extent to which each statement was true for them. The final score was reversed indicating few problems and high levels of socio-cultural competence. In the present study, Cronbach’s α was .89.

### Data Analysis Plan

A cluster analysis was performed to determine whether it was possible to distinguish different profiles of Tunisian adolescents on the basis of their acculturation outcomes (acculturation attitudes, ethnic and national identity, ethnic and national peer social contacts), and to determine whether it was possible to distinguish different profiles of Italian adolescents on the basis of their acculturation expectations (multiculturalism, melting pot, segregation, exclusion), national identity, ethnic and national peer social contacts. Chi-square analyses were carried out to determine if profile configurations were related to adolescents’ gender, length of residence and generation for Tunisian adolescents and to adolescents’ gender for Italian adolescents. In order to examine differences in psychological and sociocultural adaptation of Tunisian adolescents, and in psychological adaptation of Italian adolescents, associated to the profile configurations, a one-way MANOVA was then performed. Furthermore, a number of other MANOVAs were carried out to examine the effects of gender (for Tunisian and Italian adolescents) as well as of length of residence and generation (for Tunisian adolescents).

## Results

### Descriptive Statistics and Correlations

Means and standard deviations of, and Pearson product-moment correlation coefficients between, study variables for the Tunisian and the Italian participants are displayed in Tables 1 and 2, respectively.

**Table 1:** Means and Standard Deviations of, and Pearson Product-Moment Correlation Coefficients Between, Study Variables, for Tunisian Adolescents

Variable	1	2	3	4	5	6	7	8	9	10	11	12	13	14	15
1. Integration	-														
2. Assimilation	-.12	-													
3. Separation	-.33***	.08	-												
4. Marginalisation	-.17	.07	.07	-											
5. Ethnic social contact	.09	-.19*	.28***	-.08	-										
6. National social contact	.23**	.09	-.36***	-.09	-.14	-									
7. Ethnic identity	.18*	-.21*	.14	.05	.19*	.01	-								
8. National identity	.26**	.21*	-.37***	-.13	-.13	.49***	-.15	-							
9. Self-esteem	.22*	-.12	-.22*	.01	.01	.18*	.12	.21*	-						
10. Life satisfaction	.24**	.05	-.05	.04	.03	.16	.16	.26**	.48***	-					
11. Psychological problems	-.08	-.06	.09	.00	-.02	.02	-.02	-.02	-.52***	-.28***	-				
12. Socio-cultural adaptation	.20*	.00	-.19*	.02	-.14	.20*	.06	.23*	.48***	.30***	-.43***	-			
13. Gender	-.02	.09	.16	.11	.19*	.07	-.03	-.10	-.01	-.11	-.21*	.00	-		
14. Length of residence	.04	.03	-.25**	-.25**	-.21*	.24**	-.11	.38***	.12	.15	-.13	.15	-.08	-	
15. Generation	.07	.03	-.29***	-.21*	-.19*	.26**	-.12	.38***	.12	.15	-.16	.15	-.04	.94***	-
*M*	4.27	1.96	2.11	1.14	3.54	4.10	4.34	3.60	3.90	3.48	2.18	4.04	0.50	10.05	0.57
*SD*	0.73	0.94	0.77	0.20	1.08	1.02	0.58	1.11	0.63	0.90	0.67	0.64	0.50	5.61	0.50

*p < .05. **p < .01. ***p < .001.

**Table 2:** Means and Standard Deviations of, and Pearson Product-Moment Correlation Coefficients Between, Study Variables for Italian Adolescents

Variable	1	2	3	4	5	6	7	8	9	10	11
1. Multiculturalism											
2. Melting pot	-.04	-									
3. Segregation	-.08	.39***	-								
4. Exclusion	-.35***	.55***	.42***	-							
5. Ethnic social contact	.10	-.06	-.05	-.05	-						
6. National social contact	.21*	-.02	.05	-.07	.24**	-					
7. National identity	.03	-.16	-.09	-.14	-.04	.12	-				
8. Self-esteem	.24**	-.27**	-.24**	-.37***	.07	.15	.31***	-			
9. Life satisfaction	.19*	-.19*	-.27**	-.24**	.05	.04	.36***	.48***	-		
10. Psychological problems	.05	.11	.30***	.10	-.05	-.10	-.36***	-.53***	-.53***	-	
11. Gender	-0.01	0.18*	0.21*	0.08	0.26**	0.26**	0.04	0.22*	-0.04	-0.18*	-
*M*	4.14	1.70	1.91	1.49	1.49	4.44	3.80	4.11	3.65	2.38	.44
*SD*	0.65	0.54	0.42	0.47	0.69	0.58	0.87	0.58	0.79	0.77	.50

*p < .05. **p < .01. ***p < .001.

### Tunisian Adolescents Acculturation Profiles

We determined configurations through Ward’s (1963) clustering algorithm on the standardized acculturation process variables. The similarity between adolescents’ acculturating profiles was measured by squared Euclidean differences. The number of configurations to retain was decided by examining a scree plot of distance coefficients as a function of the number of configurations at each agglomerative step (Aldenderfer & Blashfield, 1984). Two configurations were retained because the scree plot indicated that the presence of additional configurations (more than two) did not reduce distance coefficients more than a minimal amount. Table 3 shows the number and percentage of adolescents in each configuration, along with means and standard deviations of acculturating variables, and semantic labels for the configurations. For descriptive purposes, the value of ± 0.30 was used as a cutoff to distinguish above and below average mean scores. The acculturation configurations were labelled as follows.

1. *Ethnic profile* (39.52%). Mean scores above average on separation and ethnic social contact. Mean scores below average on integration, national social contact and national identity.
2. *Integrated-national profile* (60.48%). Mean scores above average on integration, national identity and national social contact. Mean scores below average on separation.

**Table 3:** Means and Standard Deviations of Tunisian Acculturation Variables (Z-Scores) by Configuration (n = 124)

Variable	Ethnic profile (*n* = 49, 39.52%)		Integrated-National profile (*n* = 75, 60.48%)	
	*M*	*SD*	*M*	*SD*
Integration	**-0.51**	1.05	**0.36**	0.81
Assimilation	-0.09	0.91	0.04	1.07
Separation	**0.76**	0.82	**-0.51**	0.75
Marginalisation	0.13	1.05	-0.07	0.98
Ethnic social contact	**0.41**	0.63	-0.27	1.10
National social contact	**-0.81**	1.01	**0.49**	0.58
Ethnic identity	0.12	0.90	-0.03	1.03
National identity	**-0.76**	0.89	**0.48**	0.75

*Note.* Means in bold represent values above and below the cutoff value of ± 0.30.

**Figure 1:**
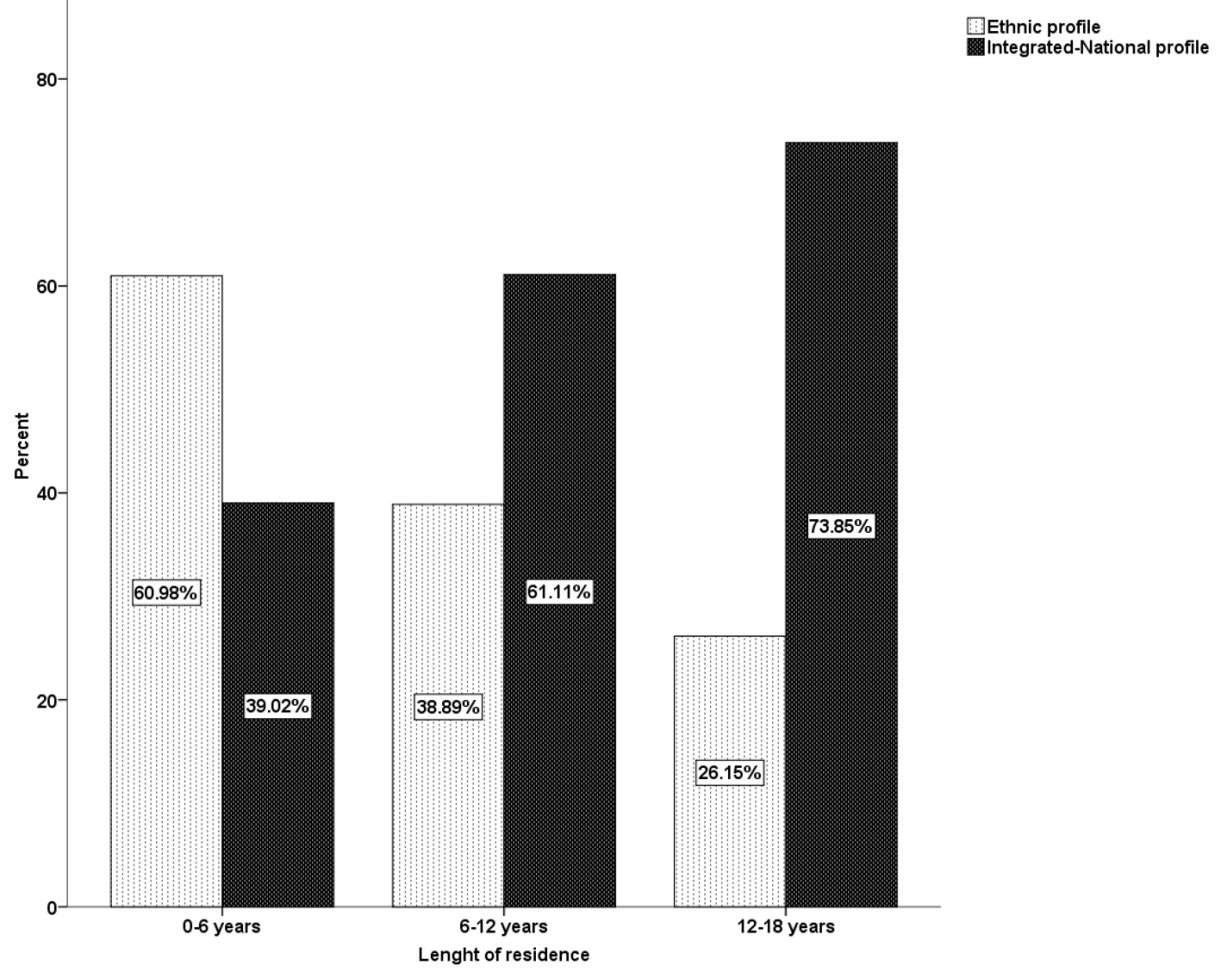
Acculturation profiles by length of residence.

Three chi-square analyses determined if configurations were related to adolescents’ gender, length of residence, and generation. First, the analysis did not indicate an association between configuration membership and gender: χ^2^(1) = 1.13, ns. Second, the two configurations showed a clear pattern of differences across the three length-of-residence categories, χ^2^(2) = 12.76, *p* < .01 (see Figure 1).

The integrated-national profile was more frequent among adolescents with longer residence; the proportion of this profile among those born in Italy or with 12 years or more of residence was almost the double that of those with 6 years or less of residence. In contrast, the ethnic profile was considerably less frequent in those with longer residence; over 60 per cent of those with 6 years or less residence showed an ethnic profile, while only about 26 per cent of those with the longest residence showed this profile. Third, the analysis indicated an association between cluster membership and generation: χ^2^(1) = 15.60, *p* < .001. Specifically, 65.31% of ethnic profile was represented by first-generation adolescents, while 70.67% of integrated-national profile was composed of second-generation youngsters. Table 4 and 5 show the number and percentage of adolescents in each configuration for first-generation and second-generation immigrant adolescents respectively, along with means and standard deviations of acculturating variables.

**Table 4:** Means and Standard Deviations of Tunisian Acculturation Variables (Z-Scores) by Configuration in First-Generation Immigrant Adolescents (n = 54)

Variable	Ethnic profile (*n* = 32, 59.26%)		Integrated-National profile (*n* = 22, 40.74%)	
	*M*	*SD*	*M*	*SD*
Integration	**-0.46**	1.19	**0.48**	0.92
Assimilation	0.04	0.96	-0.15	1.00
Separation	**0.87**	0.85	**-0.43**	0.58
Marginalisation	**0.30**	1.11	0.15	1.10
Ethnic social contact	**0.44**	0.61	-0.11	1.05
National social contact	**-0.80**	0.99	**0.42**	0.44
Ethnic identity	0.08	0.94	0.23	1.07
National identity	**-0.90**	0.88	0.24	0.81

*Note.* Means in bold represent values above and below the cutoff value of ± 0.30.

**Table 5:** Means and Standard Deviations of Tunisian Acculturation Variables (Z-Scores) by Configuration in Second-Generation Immigrant Adolescents (n = 70)

Variable	Ethnic profile (*n* = 17, 24.29%)		Integrated-National profile (*n* = 53, 75.71%)	
	*M*	*SD*	*M*	*SD*
Integration	**-0.59**	0.77	**0.31**	0.76
Assimilation	**-0.32**	0.77	0.13	1.10
Separation	**0.56**	0.75	**-0.54**	0.81
Marginalisation	-0.20	0.87	-0.16	0.92
Ethnic social contact	**0.36**	0.68	**-0.34**	1.13
National social contact	**-0.82**	1.07	**0.52**	0.63
Ethnic identity	0.21	0.86	-0.14	1.00
National identity	**-0.48**	0.86	**0.57**	0.71

*Note.* Means in bold represent values above and below the cutoff value of ± 0.30.

Maintaining the value of ± 0.30 as a useful cutoff for descriptive purposes, the levels of the mean z-scores in relation to the two generation categories differed on assimilation and marginalization for ethnic profile and on ethnic social contact and national identity for integrated-national profile. Specifically, in the first case (ethnic profile) assimilation mean z-score was below average among second-generation adolescents and marginalization mean z-score was just above average among first-generation adolescents. In the second case (integrated-national profile) ethnic social contact mean z-score was below average and national identity mean z-score was above average among second-generation adolescents.

### Italian Adolescents Acculturation Expectation Profiles

Cluster analysis was carried out with the variables associated with the host process, following the previous procedure. Two configurations were retained because the scree plot indicated that the presence of additional configurations (more than two) did not reduce distance coefficients more than a minimal amount. Table 6 shows the number and percentage of adolescents in each configuration, along with means and standard deviations of acculturation expectation variables, and semantic labels for the configurations. The acculturation expectation configurations were labelled as follows:

1. *Not-multicultural profile* (36.34%). Mean scores above average on melting pot, segregation and exclusion. Mean scores below average on multiculturalism and national social contact.
2. *Multicultural profile* (64.56%). Mean scores above average on multiculturalism. Mean scores below average on melting pot, segregation and exclusion.

A 2 (configuration) × 2 (gender) chi-square analysis did not indicate an association between configuration membership and gender: χ^2^(1) = 0.14, ns.

**Table 6:** Means and Standard Deviations of Italians Acculturation Expectation Variables (Z-Scores) by Configuration (n = 129).

Variable	Not-multicultural profile (*n* = 47, 36.34%)		Multicultural profile (*n* = 82, 63.56%)	
	*M*	*SD*	*M*	*SD*
Multiculturalism	**-0.74**	0.94	**0.43**	0.76
Melting pot	**0.52**	0.99	**-0.30**	0.88
Segregation	**0.54**	0.99	**-0.31**	0.87
Exclusion	**0.89**	0.88	**-0.51**	0.64
Ethnic social contact	-0.27	0.74	-0.15	1.10
National social contact	**-0.35**	1.31	-0.20	0.70
National identity	-0.15	1.06	0.08	0.96

*Note.* Means in bold represent values above and below the cutoff value of ± 0.30.

### Relations Between Acculturation Profiles and Tunisian Adolescents’ Adaptation

In order to examine differences in psychological and sociocultural adaptation associated to cluster membership among Tunisian adolescents, a one-way MANOVA was performed. There was a significant multivariate effect, Wilks’ Lambda = .81, *F*(4,117) = 6.74, *p* < .001, η^2^ = .19. Univariate ANOVAs revealed significant effects for self-esteem, life satisfaction, and socio-cultural competence. Results are displayed in Table 7. Tunisian adolescents belonging to the integrated-national profile showed higher levels of self-esteem, life satisfaction and socio-cultural competence than those belonging to the ethnic profile. In the same way, we also examined how well immigrant youth were adapting as a function of gender, length of residence, and generation, using three different one-way MANOVA with the four adaptation scores as dependent variables. There was only a significant multivariate effect for gender, Wilks’ Lambda = .92, *F*(4,120) = 2.65, *p* < .05, η^2^ = .08. Univariate ANOVAs revealed significant effects for psychological problems, *F*(1,123) = 5.14, *p* < .05, η^2^ = .04, with girls (*M* = 2.30, *SD* = 0.66) having higher scores than boys (*M* = 2.04, *SD* = 0.62).

**Table 7:** Means and Standard Deviations of Tunisian Adolescents’ Adaptation Variables

Variable	Ethnic profile		Integrated-National profile		*F* (1,120)	η^2^
	*M*	*SD*	*M*	*SD*		
Self-esteem	3.71	0.65	4.00	0.58	6.99**	.05
Life satisfaction	3.27	0.96	3.62	0.84	4.51*	.04
Psychological problems	2.13	0.55	2.19	0.71	0.28	.00
Socio-cultural adaptation	3.78	0.68	4.20	0.55	14.17***	.11

*p < .05. **p < .01. ***p < .001.

### Relationships Between Acculturation Expectation Profiles and Italian Adolescents’ Adaptation

In order to examine differences in psychological adaptation associated to cluster membership among Italian adolescents, a one-way MANOVA was performed. There was a significant multivariate effect, Wilks’ Lambda = .83, *F*(3,125) = 8.38, *p* < .001, η^2^ = .17. Univariate ANOVAs revealed significant effects for self-esteem and life satisfaction. Results are displayed in Table 8. Italian adolescents belonging to the multicultural profile showed higher levels of self-esteem and life satisfaction than those belonging to the not-multicultural profile. In the same way, we also examined how well autochthonous youth were adapting as a function of gender. There was a significant multivariate effect, Wilks’ Lambda = .90, *F*(3,125) = 4.61, *p* < .01, η^2^ = .10. Univariate ANOVAs revealed significant effects for self-esteem, *F*(1,127) = 6.58, *p* < .05, η^2^ = .05, and psychological problems, *F*(1,127) = 4.15, *p* < .05, η^2^ = .03. Italian boys (*M* = 4.28, *SD* = 0.58) showed higher levels of self-esteem than girls (*M* = 4.01, *SD* = 0.56). In contrast, Italian girls (*M* = 2.48, *SD* = 0.81) showed higher levels of psychological problems than boys (*M* = 2.20, *SD* = 0.64).

**Table 8:** Means and Standard Deviations of Italian Adolescents’ Adaptation Variables

Variable	Not-multicultural profile		Multicultural profile		*F* (1,127)	η^2^
	*M*	*SD*	*M*	*SD*		
Self-esteem	3.85	0.56	4.25	0.53	16.23***	.11
Life satisfaction	3.36	0.68	3.81	0.81	10.28***	.07
Psychological problems	2.45	0.76	2.35	0.77	0.45	.00

***p < .001.

## Discussion

The general purpose of the present study was to analyze emerging intercultural profiles in relation to adaptation of immigrant and autochthonous adolescents living in Sicily (Italy). Our results show that both immigrant and autochthonous adolescents can be divided into two groups or profiles which are associated to different psychosocial outcomes.

The first specific goal was to identify some acculturation profiles among Tunisian adolescents. Our hypothesis was only partially supported since the findings produced two acculturation profiles, rather than four. One profile was named ethnic (39.52% of the sample) and it was characterized by a strong preference towards separation as acculturation strategy, and by a predilection for maintaining social contacts with people of the same ethnic group. The second profile was called integrated-national (60.48% of the sample) and it was characterized by a preference towards integration as acculturation strategy, a strong identification with autochthonous group (Italians) and a preference towards social contact with Italians. Moreover, it is interesting to note that ethnic profile seems to be prevalent among first-generation immigrants, whereas adolescents of the integrated-national profile are prevalently second-generation immigrants. This is also confirmed by the results related to the association between acculturation profiles and adolescents’ length of residence because the ethnic profile prevailed in immigrants with a lower length of residence while the integrated-national profile was prevailing in adolescents who lived in the society of settlement from their birth or from their early school years on. Thus, the more extended is the period of stay in the new country, the more immigrant adolescents tend to establish ties with the host country culture. This is particular evident for adolescents of the integrated-national profile because our findings showed that second-generation immigrants reported less social contacts with ethnic peers and an higher national identity than first-generation immigrants. Perhaps, it could be hypothesized the existence of a developmental and adaptive process that goes from the prevalence of separation and ethnic social contact to a declarative form of integration along with a behavioural form of assimilation consisting prevalently of national social contact. This is in line with the results of Schwartz and Zamboanga (2008) showing that second-generation immigrants are more likely to evidence a combination of assimilation and integration, and first-generation immigrants are more likely to be classified as separated.

In a certain way, we provided evidence that acculturation is a very complex process that, despite some general features, needs to be analyzed in relation to the context in which takes place (e.g., Chirkov, 2009). The use of a priori classification rule assumes that all four categories (integration, separation, assimilation, and marginalization) exist and are equally valid (Rudmin, 2003). However, our findings as well as those of other authors which used empirical rigorous ways of classifying individuals (Schwartz et al., 2010; Schwartz & Zamboanga, 2008) suggest that not all of Berry’s categories may exist in a given sample or population. In particular, we found evidence for the existence of only two categories of four, one mixing some features of integration and assimilation and the other resembling separation. These results can be better understood on the light of the features of two considerations about the research context. On the one hand, the migratory flows towards Sicily are a relatively new phenomenon, typical of the last years, so it is likely that the acculturation process produce less differentiated outcomes than in other countries that are multicultural/plural from decades (e.g., Canada, USA). On the other hand, the evident presence of an ethnic profile can be justified considering that Tunisians living in Sicily were able to keep strong ties with their own heritage culture as it is shown by the presence of many Tunisian institutions (schools, places of worship, community centres, and restaurants) that are likely to contribute to the maintenance of their habits.

The last important consideration about the acculturation profiles is related to the absence of a cluster resembling marginalization. This is in line with other studies using empirically based clustering methods that have found small or nonexistent marginalization groups (e.g., Schwartz et al., 2010; Schwartz & Zamboanga, 2008) and it is consistent with what argued by Del Pilar and Udasco (2004) and Rudmin (2003), who consider implausible for someone to develop a cultural sense of self without drawing on either the heritage or receiving cultural contexts.

The second specific goal of the study was to identify some acculturation expectation profiles among autochthonous adolescents. The results confirmed our prediction that two acculturation expectation profiles could be identified. The not-multicultural profile (36.34%) was characterized by preferences towards melting pot, segregation and exclusion as acculturation expectations, and a general negative attitude towards multiculturalism. The multicultural profile (63.56% of the sample) was characterized by a positive attitude towards multiculturalism and low levels of melting pot, segregation and exclusion.

These results are in line with the existing literature (e.g., Arends-Tóth & Van de Vijver, 2003; Berry, 2011) indicating that acculturation expectations can be considered as an unidimensional construct with preference for multiculturalism anchoring one end of the dimension, and the preferences for melting pot, segregation, and exclusion, anchoring the other end. According to the authors, this unidimensional structure is likely to result in contexts where the attitudes are very positive for integration, and there is a common rejection of the other three ways. This explanation can be appropriate for the context of the research because Sicily is characterized by high rates of openness towards cultural diversity, especially towards Tunisian people who live on the other side of Mediterranean sea very close to Sicily. As a matter of fact, Sicilians and Tunisians have a long tradition of exchanges and of positive contacts that can promote positive intergroup attitudes (Berry, 2013; Lebedeva & Tatarko, 2013).

The third specific goal was to analyze the relationships between acculturation profiles and adaptation of the Tunisian adolescents in terms of psychological (self-esteem, life satisfaction, and psychological problems) and sociocultural adaptation. Our findings provided some empiric evidence for integration hypothesis (Berry, 2013) stating that integration has a significant and positive relationship with both psychological and sociocultural adaptation. Adolescents in the integrated-national profile showed higher levels of self-esteem, life satisfaction and sociocultural competence than those in the ethnic profile, but no significant differences were found with regard to the scores of psychological problems. In general, the present study matches with the others that report a positive association between a favourable attitude towards integration and adolescents’ adaptation, underlining the psychological relevance of the opportunities to both maintain the original culture and to be in contact with the culture of the host country (for a review, Nguyen & Benet-Martínez, 2013).

The fourth goal was to investigate the relationships between the acculturation expectation profiles and the psychological adaptation of the autochthonous adolescents. Our results show that adolescents who belong to multicultural profile showed higher levels of self-esteem and life satisfaction when compared to their not-multicultural counterpart. This is in line with previous studies on similar topics (e.g., Verkuyten, 2009) which have observed that recognition and acceptance of cultural diversity appears to be associated with better self-feelings of majority group members. Thus, the results seem to support the idea that multicultural acceptance is related to the psychological well-being of majority group.

To sum up, our study has interesting implications for the research in this topic as well as providing useful suggestions to elaborate settlement policies in Italy. First, the findings advocate for a position midway between Berry’s model (Berry, 2013) and some of the criticisms addressed to it (e.g., Rudmin, 2009; Schwartz et al., 2010; Schwartz & Zamboanga, 2008) using clustering techniques. The findings of our study lend support to the use of clustering methods as a way of including multiple indicators of acculturation, thereby gaining a more comprehensive understanding of acculturation. By this means research can overcome a “one size fits all” approach (Rudmin, 2003) in order to adopt a more realistic approach with a more explanatory power. Second, the study takes into account intercultural profiles of both immigrant and autochthonous adolescents as it is part of the MIRIPS project. However, it does not consider yet the mutual interactions between the two groups since they are analyzed in a separate way. Future studies need to focus more on the dynamic and interactional nature of the acculturation and intercultural relations. Moreover, the study provides some evidence for the integration hypothesis with regard to the Tunisian adolescents living in Sicily, a group that has not already been considered by the scholars. Our findings highlight also the importance of considering the relationships between intercultural profiles and adaptation in the members of dominant groups. These associations were neglected by previous research that was mainly focused on the effect of acculturation on immigrants’ adaptation. Finally, the results of the study support the idea that social policies have to pursue the integration of immigrant adolescents providing them with opportunities to maintain their culture of origin and, concurrently, to participate at the larger societies and to have contacts with autochthonous people. At the same time, autochthonous adolescents have to be provided with more opportunities to develop a positive attitude towards multiculturalism, for instance, through intercultural education programs.

However, our results should be interpreted in light of some limitations. First, the study involved a small sample size that needs to be enlarged in further researches. Such a limited sample size did not allow us to perform a replication of cluster analysis splitting Tunisian participants in first- and second-generation immigrants. Similarly, we could not perform neither a 2 (profile) x 2 (gender) x 3 (length of residence) x 2 (generation) MANOVA to examine the relations between acculturation profiles and Tunisian adolescents’ adaptation nor a 2 (profile) x 2 (gender) MANOVA in order to examine the relations between acculturation expectation profiles and Italian adolescents’ adaptation. Second, the study was focused only on a specific group of immigrants (Tunisians) that is the most representative in the context of the research. However, it would be useful to extend the study to the analysis of other cultural groups living in Sicily, such as people from China, Bangladesh, Romania, and Central Africa. Third, more attention to the developmental processes underlying the formation of intercultural profiles during adolescence has to be spent through the use of longitudinal design. Fourth, it would be interesting to set up further studies aimed to shed some light on the dynamic process in which perceptions of dominant group members’ preferences have an impact on non-dominant group members’ acculturation attitudes, as well as on the quality of intergroup relations itself (Brown & Zagefka, 2011). Fifth, there is the need to integrate quantitative methods already used by the researchers in this field with qualitative methods, such as in-deep interviews and focus groups in order to better understand this complex phenomena.
